# Prevalence and overlap of known undernutrition risk factors in children in Nairobi Kenya

**DOI:** 10.1111/mcn.13261

**Published:** 2021-08-06

**Authors:** Antonina N. Mutoro, Ada L. Garcia, Elizabeth W. Kimani‐Murage, Charlotte M. Wright

**Affiliations:** ^1^ Human Nutrition, School of Medicine, Dentistry and Nursing, College of Medical, Veterinary and Life Sciences University of Glasgow Glasgow UK; ^2^ Maternal and Child Wellbeing Unit African Population and Health Research Center Nairobi Kenya; ^3^ Child Health, School of Medicine University of Glasgow Glasgow UK

**Keywords:** children, risk, stunting, undernutrition, wasting

## Abstract

We aimed to describe the co‐occurrence of known risk factors for undernutrition and the prevalence of modifiable risks in wasted, stunted and healthy children. Quota sampling was used to recruit healthy [weight for age *Z* scores (WAZ) > −2 SD] and undernourished [weight for length (WLZ) or WAZ scores ≤ −2 SD] children aged 6–24 months from seven clinics in low‐income areas of Nairobi. Structured interviews were used to identify exposure to socioeconomic, water and hygiene, infant feeding, dietary and behavioural risks (low interest in food, high food refusal and force feeding). We recruited 92 wasted WLZ ≤ −2 SD, 133 stunted (length for age *Z* scores LAZ ≤ −2 SD) and 172 healthy (LAZ and WLZ > 2SD) children. Nearly all children were exposed to hygiene risks (90%) and low dietary diversity (95%) regardless of nutritional status. Stunted children were more likely to be exposed to socio‐economic risks (54% healthy, 64% wasted and 72% stunted; *P* = 0.001). Compared with healthy children, wasted and stunted children were more likely to be exposed to infant feeding (25% healthy, 40% wasted and 41% stunted; *P* = 0.02) and behaviour risks (24% healthy, 49% wasted, and 44% stunted; *P* = 0.004). Overall, wasted and stunted children were twice as likely to be exposed to more than three risks (23% healthy, 48% wasted, and 50% stunted; *P* = <0.001). They were also more likely to be exposed to more than three modifiable risks (dietary, handwashing and behaviour risks). Wasting and stunting are associated with exposure to multiple risk factors, many of which are potentially modifiable using targeted advice.

**Key messages**

- Most of the undernourished children were exposed to feeding issues such as problems with breast feeding, late complementary feeding, low meal frequency and food refusal, which have the potential to be modified via advice and support.
- Undernourished children were exposed to a variable number and combination of risk factors, which suggests that an individualized problem‐solving treatment approach is required.

## 1 BACKGROUND

Child undernutrition remains a public health problem in many low‐ and middle‐income countries (LMICs), despite some global improvement (Global Nutrition Report, 2020). Undernourished children are classified as either wasted or stunted based on their anthropometric measurements and tend to be managed differently, yet both conditions may be present in the same child and may coexist within communities (Kimani‐Murage et al., 2015; Myatt et al., 2018; Olofin et al., 2013). A better understanding of the common causes of both child wasting and stunting is therefore required to inform the design of effective prevention and treatment interventions.

Many population‐based studies have assessed risk factors of undernutrition (Lohia & Udipi, 2014; Moursi et al., 2009; Ruel & Menon, 2002), but less is known about how these risk factors overlap in individual undernourished children and the extent to which each may be modifiable. For example, a child presenting with dietary risk factors, which can potentially be modified using behaviour change interventions administered in health facilities by health care workers, is also likely to be exposed to other social and childcare risks (Chisti et al., 2007). Risk factor studies commonly focus on rural populations in LMICs; yet in urban settings, a high proportion resides in slum settlements, where undernutrition rates are high. For example, in Nairobi, close to 60% of the population resides in slums, and close to 50% of children in these slums are stunted (Concern Worldwide, 2017; Kimani‐Murage et al., 2015).

This study therefore aimed to describe the extent of co‐occurrence of known risk factors for undernutrition in wasted and stunted children aged 6–24 months in slum areas in Nairobi, and the prevalence of those that might be individually modifiable. The specific objectives were to (1) create risk scores for different groups of risk factors, (2) assess the relative prevalence of these risk factors in wasted and stunted children compared with healthy children and (3) assess the extent to which the risk factors overlap in undernourished children.

## 2 METHODS

### 2.1 Study design and setting

This cross‐sectional study was conducted in seven out of 80 health facilities in Nairobi: Mbagathi District hospital, Kayole II sub‐county hospital, Ruben Medical Clinic, Makadara health centre, Mukuru kwa Njenga health centre, Soweto PhC clinic, which run child welfare clinics, outpatient therapeutic and supplementary feeding programmes. The health facilities were selected because of their proximity to slum areas, which are characterized by extreme poverty levels, poor access to basic hygiene and sanitation facilities as well as poor health outcomes (African Population and Health Research Center, 2014).

### 2.2 Sampling, inclusion and exclusion criteria

Quota sampling was used to recruit caregiver child pairs aged 6–24 months based on their nutrition status (healthy vs. undernourished). Undernourished children were further recruited based on the severity of their undernutrition and treatment status. Undernourished children were recruited between February and July 2015. At that time, children were included with an aim to recruit equal numbers of moderate and severely malnourished children based on their treatment status that is whether they had started treatment with ready to use foods (Mutoro et al., 2020). Children with either weight for age (WAZ) or weight for length (WLZ) *Z* scores ≤ −2 standard deviations (SD) were recruited initially as well as any other child with severe stunting (length for age *Z* scores, LAZ ≤ −3 SD). Children were excluded if they either had medical complications such as oedema (n = 2), other medical conditions such as congenital heart disease (*n* = 1) or cleft lip and palate (*n* = 1). Healthy children were recruited between July and August 2016 in the child welfare clinics in the same centres and were included if they had no major medical conditions and had a WAZ > −2 SD. The healthy group was an ideal control because it would enable us to tease out what other factors potentially increase the risk of childhood undernutrition especially in cases where healthy and undernourished children are exposed to similar environmental conditions.

For this analysis, we aimed to compare wasted or stunted children with healthy so the sample was divided into three groups. Firstly, children were defined as wasted if they had WLZ score ≤ −2 SD. Children who were both wasted and stunted (WLZ ≤ −2 and LAZ ≤ −2) were also classified as wasted (WaSt) because their risk of mortality is equivalent to that of children who are severely wasted (Myatt et al., 2018). Then, a stunted only group was defined as any child with LAZ ≤ −2 SD, but WLZ > −2 SD. Finally, the healthy group was defined as any child with WLZ, WAZ and LAZ > −2 SD. Children who had a low, WAZ ≤ −2, but WLZ and HAZ > −2 SD were excluded from the study.

### 2.3 Anthropometry

Anthropometric measurements were taken using standardized measurements (Lohman et al., 1992; World Health Organization, 2008b). Weight was measured using a digital weighing scale (SECA 385 digital weighing scale III) to the nearest 0.1 kg. Supine length was measured to the nearest 0.1 cm using a portable Rollameter (Raven Equipment Ltd Dunmow, U.K) or a UNICEF length board. Mid upper arm circumference was measured using MUAC tapes (S0145620 MUAC, Child 11.5 Red/PAC‐50) to the nearest 0.1 cm.

### 2.4 Measures used to assess risk

#### 2.4.1 Socio‐economic and hygiene characteristics

A structured interview schedule was used to collect risk factor data grouped into five categories: socio‐economic, hygiene, infant feeding, diet, eating and feeding behaviour.

Three variables, ownership of a TV or radio, number of children under 5 years and parental education, were used as socio‐economic risk markers as described in Table 1. The variables were selected because they are associated with child nutrition status (Abuya et al., 2012; Checkley et al., 2004; Victora et al., 1986).

**Table 1:** Measures used to assess childcare risk factors

	Components	Scores assigned	At risk: Score 1
		No risk: Score 0	
Socio‐economic risks	Parental education Household composition	Score 0 if all of following present: no television, no radio, parents educated only to primary level, more than one child under 5 years	Score 1 if otherwise
Hygiene risk	Caregiver handwashing	Score 0 if all of following done: after using toilet, after changing baby, before feeding child or eating, before preparing food	Score 1 if otherwise
	Source of water for household use	Score 0 if piped into house	Score 1 if public tap or another source
	Toilet ownership	Score 0 if owned by household	Score 1 if shared
Infant feeding	Breastfeeding status	Score 0 if currently breastfeeding	Score 1 if child is not currently breastfeeding
Complementary feeding	Introduction of complementary foods	Score 0 for timely introduction at 6 months	Score 1 for early (<6 months) or late (>6 months) introduction
Dietary and feeding practices	Dietary diversity‐ 8 food groups meat/fish/poultry/organ meats, eggs, dairy (breast milk not included), legumes (beans, lentils, mung beans, and dried peas), fruits and leafy vegetables, oil/fats/margarine, starchy staples	Score 0 if offered at least 4 food groups	Score 1 if offered <4 food groups
	Plated meal frequency	Score 0 if offered 2 or more meals aged 6–12 months, 3 or more 12–24 months	Score 1 if child is 6–12 months and is offered <2 meals, 12–24 months <3 meals
Eating and feeding behaviour	Interest in food, food refusal (avoidance) score and force feeding score	Score 0 if child has low avoidance, high interest in food and low force feeding	Score 1 if otherwise

*Note*: For each risk area, high risk is defined as any score > 0.

Hygiene risk was assessed using three variables: main source of water for household use, toilet ownership and caregivers' handwashing practices (Table 1). Handwashing has important health benefits including prevention of diarrhoea (Billig et al., 1999; Nizame et al., 2013). Mention of handwashing during key times: after using the toilet, after changing the baby's nappy, before handling and preparing food, before feeding the child was therefore used to assess personal hygiene (Billig et al., 1999). Source of water for household use was transformed into two categories piped into household versus other sources, based on the hypothesis that caregivers who had piped water in their houses had access to more water, which enhances their hygiene practices (Billig et al., 1999). For each variable, caregivers who had positive attributes for example owned their own toilet and household assets scored 0 while those without scored 1.

Infant feeding practices was assessed using two variables: timing of introduction of complementary foods and breastfeeding status (Table 1). Timing of introduction of complementary foods was recoded into two groups reflecting timely introduction (at 6 months) and early/late (either below 6 months or above months).

The dietary risk group included two items: low dietary diversity and low meal frequency. Dietary diversity was assessed using a short food frequency table, based on World Health Organization (WHO) principles applied to locally use complementary foods, grouped into eight categories (Table 1) with seven possible responses from never/rarely to more than once daily. Food groups eaten rarely and weekly were scored 1 while those eaten daily were scored 0. The number of food groups eaten daily was counted to determine adequacy. Low dietary diversity was defined as less than four food groups offered daily, based on WHO standards (World Health Organization, 2008a).

Overall, meal frequency was assessed by asking caregivers to report types of food offered within each of five parts of the previous day. Foods were classified as either plated foods, finger foods or drinks. Plated foods were described as any cooked foods served on a plate. Finger foods included foods that a child could pick and self‐feed, such as pieces of fruit, biscuits and bread. Drinks included thin porridge, yoghurt and tea. Porridge was classified as a drink because in Kenya porridge has a liquid consistency and is low in energy (Kulwa et al., 2015; Michaelsen et al., 2009; Treche & Mbome, 1999). A summary score was created by adding up the total number of plated meals and snacks. Drinks were excluded because feeding frequency includes only nonliquid foods (PAHO, 2003). Age‐specific WHO meal frequency recommendations were then used to define adequacy by age (Table 1).

Eating and feeding behaviour (EFB) included three items avidity, food refusal and force‐feeding, assessed using a set of structured questions, some of which were derived from the Gateshead Millennium Study in the United Kingdom (Mutoro et al., 2020). Details on how avidity, food refusal and force‐feeding variables were created are presented elsewhere (Mutoro et al., 2020). High risk behaviours were then defined as low avidity, high food refusal and high force‐feeding all of which were scored 1 if present and 0 if absent (Table 1).

The diet and EFB group items were all defined as being potentially modifiable at an individual level, as well as handwashing practices, giving a possible total of six individually modifiable risk factors.

#### 2.4.2 Sample size and data analysis

We aimed for a sample size sufficient (80% power, alpha 0.05) to detect a prevalence of 15% for any risk factor in one group compared with 30% in another (relative risk = 2) which would require 150 subjects in each of the three subgroups.

Data analysis was done using Statistical Package for Social Sciences (SPSS) version 22. For each risk category (socio‐economic, hygiene, infant feeding, dietary and behaviour), the total number of risks were counted per child. Chi‐squared analysis was used to test associations in categorical variables where Pearson's chi‐squared test was used for binary variables. The level of significance was set at *P* < 0.05.

### 2.5 Ethical considerations

Ethical approval for the study was obtained from the University of Glasgow Ethics review committee (200140057), University of Nairobi and Kenyatta National Hospital Ethics Review committee (P651/11/2014). Access to health facilities was granted by the Nairobi county and sub‐county health offices.

## 3 RESULTS

**Figure 1:**
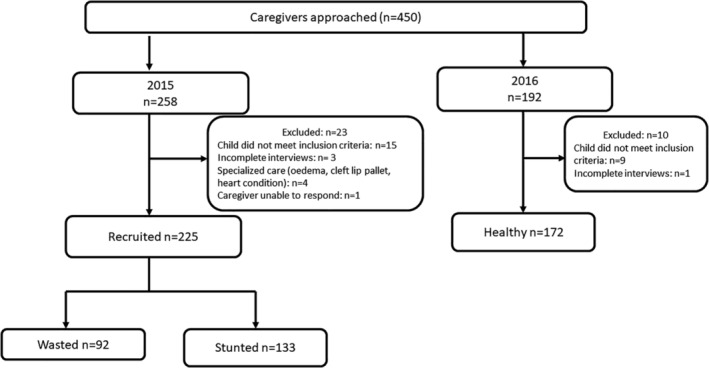
Participant recruitment study flow diagram

In total, 450 caregivers were approached for interviews, and 415 were interviewed, of whom 397 were included in the analysis (Figure 1). There were 172 healthy, 92 wasted and 133 stunted children (Table 2). Socio‐economic characteristics of study participants are presented in Table S1.

**Table 2:** Age and anthropometric characteristics by child nutrition status

	Healthy (*n* = 172)	Wasted^a^ (*n* = 92)	Stunted only (*n* = 133)
Age (months)	9.65 [8.4 to 13]	10.1 [8.9 to 14]	10.7 [8.9 to 16]
WAZ	−0.28 [−0.8 to 0.6]	−2.55 [−3.0 to −2.1]	−2.95 [−3.6 to −2.4]
WLZ	0.70 [−0.7 to 0.9]	−2.66 [−3.3 to −2.3]	−1.93 [−2.5 to −1.1]
LAZ	−0.41 [−1.1 to 0.3]	−1.07 [−1.6 to −0.5]	−2.81 [−3.6 to −2.4]

*Note*: Values are median (IQR).

Abbreviations: LAZ, length for age *Z* scores; WAZ, weight for age *Z* scores; WLZ, weight for length *Z* scores.

^a^ Includes children who are both wasted and stunted.

Over half of healthy children had one or more socio‐economic risks, rising to almost three quarters for stunted children. WaSt children had slightly higher social risk than healthy, but this was not significant (Table 3). All children had at least one hygiene risk, and 90% had more than one risk factor, with no association between the number of hygiene risks and child nutrition status (Table 3).

**Table 3:** Social, hygiene, infant feeding, dietary factors and feeding practice scores by child nutrition status

Care practices	Healthy (*n* = 172)	Wasted^a^ (*n* = 92)		Stunted (*n* = 133)	
		*n* (%)	Relative risk^b^	*n* (%)	Relative risk^b^
Total socio‐economic risks^c^					
*n* (%) with no risk	76 (46.3)	33 (35.9)	1.19 [0.9 to 1.6]	37 (28.0)	1.34 [1.1 to 1.6]
*n* (%) with 1 or more	88 (53.7)	59 (64.1)		95 (72.0)	
*P* compared with healthy		0.104	0.094	0.001	0.001
Total hygiene risks					
*n* (%) with 0–1 risks	17 (10.0)	9 (9.9)	1.00 [0.9 to 1.1]	11 (8.3)	1.01 [0.9 to 1.1]
*n* (%) with more than 1 risk	153 (90.0)	82 (90.1)		122 (91.7)	
*P* compared with healthy		0.977	0.974	0.606	0.602
Total hygiene risks (without handwashing)					
*n* (%) with no risk	14 (8.1)	8 (8.7)	0.99 [0.9 to 1.1]	10 (7.5)	1.01 [0.9 to 1.1]
*n* (%) with 1 or more risk	158 (91.9)	84 (91.3)		123 (92.5)	
*P* compared with healthy		0.876	0.877	0.842	0.84
Total infant feeding risks					
No risks	129 (75.0)	56 (60.9)	1.56 [1.1 to 2.3]	77 (59.2)	1.63 [1.2 to 2.3]
1 or more	43 (25.0)	36 (39.1)		53 (40.8)	
*P* compared with healthy		0.017	0.015	0.004	0.003
Total dietary risks					
No risks	5 (2.9)	7 (7.6)	0.95 [0.9 to 1.0]	6 (4.6)	0.98 [0.9 to 1.0]
1 or more	167 (97.1)	85 (92.4)		124 (95.4)	
*P* compared with healthy		0.08	0.13	0.43	0.447
Total EFB risks					
No risks	130 (75.6)	47 (51.1)	2.09 [1.5 to 2.9]	75 (56.4)	1.78 [1.3 to 2.5]
1 or more	42 (24.4)	45 (48.9)		58 (43.6)	
*P* compared with healthy		<0.001	<0.001	<0.001	<0.001

^a^ Includes children who are both wasted and stunted.

^b^ Compared with healthy.

^**c**^ Healthy children *n* = 164; eight children were excluded because did not have complete information.

Only a quarter of healthy children had one or more infant feeding risk, but this rose to 40% for children who were either WaSt or stunted and this result remained significant after adjusting for the child's age and socio‐economic risks (Table 3).

Almost all children had one or more dietary risk, with 91% healthy children having low dietary diversity, and 72% having a low meal frequency (Table S2). There was no association between child nutrition status and dietary diversity and feeding frequency. This result remained the same after children on supplements, *n* = 90, were excluded from the analysis (Table 3). Overall, a quarter of healthy children had one or more eating behaviour risks, but this rose to 49% for WaSt and 44% for stunted children (Table 3).

Overall, all children were exposed to a risk in at least one of the five risk groups (Table 4), but WaSt and stunted children were twice as likely to be exposed to four or more (23% healthy, 48% WaSt, 50% stunted *P* < 0.0001, χ^2^). All children were exposed to at least one individually modifiable risk factors, and three quarters were exposed to three or more, but WaSt children were 34% and stunted 32% more likely to be exposed to four or more; 1.2% healthy, 2.2% WaSt and 3.0% stunted children were exposed to all six modifiable risk factors.

**Table 4:** Total number of risks and individually modifiable risks by child nutrition status

Risk factors	Healthy	Wasted^a^		Stunted only	
Number of risk groups present per child	*n* (%)	*n* (%)	Relative risk^c^	*n* (%)	Relative risk^c^
1–2 risks	47 (27.3)	15 (16.3)	1.15 [1.0 to 1.3]	22 (16.5)	1.15 [1.0 to 1.3]
3 risks	85 (49.4)	33 (35.9)		45 (33.8)	
4–5 risks	40 (23.3)	44 (47.8)		66 (49.6)	
*P* chi‐squared compared with healthy		*P* < 0.001	0.031	*P* < 0.001	0.022
Number of modifiable ^b^feeding, dietary and handwashing risks					
1–2 risks	54 (31.4)	22 (24.2)	1.11 [0.9 to 1.3]	34 (25.6)	1.08 [0.9 to 1.3]
3 risks	84 (48.8)	38 (41.8)		56 (42.1)	
4–6 risks	34 (19.8)	31 (34.0)		43 (32.3)	
*P* chi‐squared compared with healthy		0.036	0.202	0.043	0.259

^a^ Includes children who are both wasted and stunted.

^**b**^ Meal frequency, dietary diversity, force feeding, avidity and food refusal.

^**c**^ Compared with healthy.

**Figure 2:**
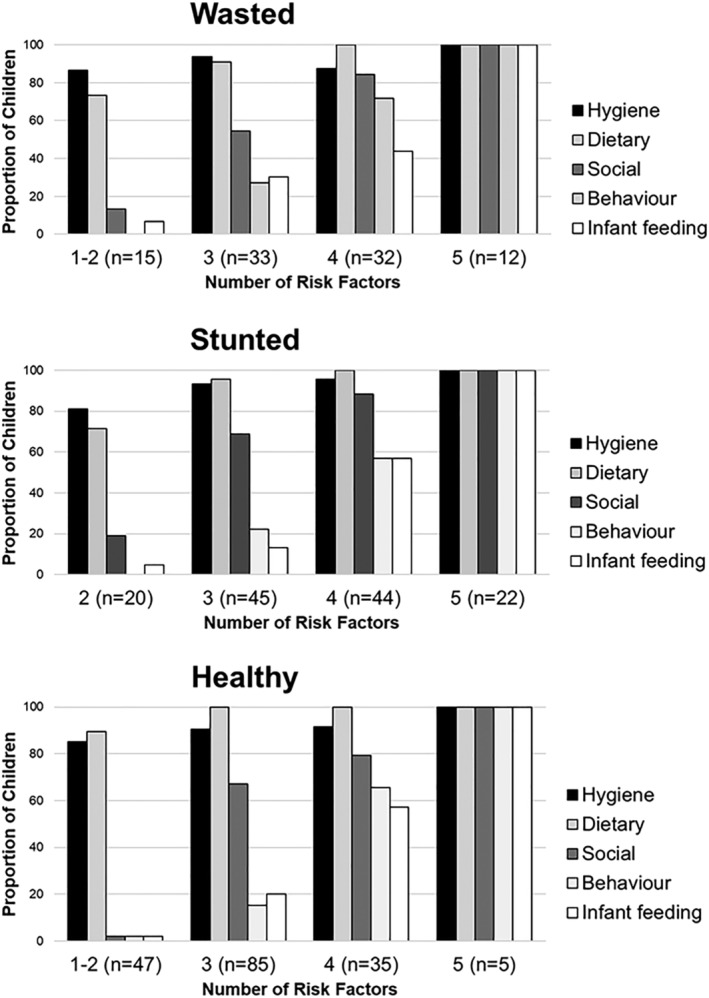
Graphs showing the number and types of risk factors in healthy, wasted and stunted children. Hygiene and dietary risks were common regardless child nutrition status and the number of risks children. Social and behaviour risks were common in children who had three or more risks

Hygiene was the predominant risk factor seen where a child exposed to only one risk type, with dietary factors where there were two, whereas the other risk factors were commonly seen only when the child was exposed to multiple factors (Figure 2). There was no association between child nutrition state (WaSt and stunting) and the risk categories.

## 4 DISCUSSION

This study aimed to identify the co‐occurrence and the number of modifiable risk factors for undernutrition, in order to better plan approaches to the care of individual undernourished children, as well as understand optimal prevention approaches. We found that nearly all children were exposed to dietary and hygiene risks regardless of their nutrition status. WaSt and stunted children were, however, more likely to have infant feeding risks and EFB risks and were more likely to be exposed to more than three risk factors.

Quota sampling was used to ensure a large sample of undernourished children was recruited from selected health facilities in urban slums in Nairobi. The study was therefore not entirely representative of children in Nairobi slums. However, recruiting from multiple health facilities enabled sampling from different slum areas and a relatively more diverse sample of caregivers. Where we can compare our findings with population surveys, they are broadly similar (Kenya National Bureau of Statistics, 2015).

These findings inform our understanding of undernutrition in early childhood, but it cannot be assumed that any risk factors play a direct causal role. They support the general model that suggests that undernutrition is not usually the result of specific exposure, so much as a cumulative exposure to general risk factors (Black et al., 2008). What these findings illustrate is how common these exposures still are in an urban slum population in a middle‐income country. Generally, the undernourished children were exposed to more risks in total, but still 2.9% of healthy children were exposed to all five and yet were not undernourished, suggesting that some individual children are inherently more vulnerable than others. Positive deviant studies in this environment could help identify protective childcare practices in healthy children, which can then be used to design community interventions targeting undernourished children (Mackintosh et al., 2002).

Wasting and stunting showed similar associations to the number and type of risk factors, except that stunting appeared be more common in children from poor socio‐economic backgrounds, a finding that has been previously reported (Black et al., 2013; Harding et al., 2018; Kenya National Bureau of Statistics, 2015; Menon et al., 2000; Mohsena et al., 2010). This suggests that whether a child wastes or stunts in response to risk exposure may also be a matter of individual vulnerability, rather than the severity or duration of exposure.

It has been rightly argued that interventions should take into account the whole range of risks identified here. However, the approach needed depends upon the specific risk as we observe in our findings. In this population, hygiene risks were ubiquitous and thus would need to be addressed at a community level. Water sanitation and hygiene (WASH) interventions when implemented alone or in combination with nutrition interventions have so far been shown to have only marginal effects on child growth and health (Humphrey et al., 2019; Luby et al., 2018). However, compliance with WASH interventions has been shown to be low in studies conducted in rural areas (Humphrey et al., 2019; Null et al., 2018), and it can be argued that without substantial infrastructure changes WASH interventions are unlikely to succeed (Cumming & Cairncross, 2016).

Low dietary diversity and infrequent meals were also ubiquitous. The measure of dietary diversity used, though based on WHO principles, had not been formally validated, but these findings were plausible, given other survey findings that indicate that a large proportion of children in Kenya do not receive diverse diets (Kenya National Bureau of Statistics, 2015). In an urban environment, there is usually no absolute food shortage, so this more likely reflects a mismatch between low income and the higher cost of a diverse diet. In this study, information on food security was not collected, but other studies have shown that food insecurity is common in Nairobi slums (Kimani‐Murage et al., 2014; Mutisya et al., 2015). It could be argued that only money transfer or income generating programmes can hope to impact sustainably on this at population level (Mohanty, 2013), given that childhood undernutrition is associated with socio‐economic inequalities (Global Nutrition Report, 2020; Mutisya et al., 2015).

Current interventions in low‐income areas tend to rely on the provision of supplementary foods, which are expensive (Heckert et al., 2019) and produce modest improvements (Das et al., 2019; Ramakrishnan et al., 2009). They also fail to identify or address modifiable dietary or behavioural risk factors, so that subsequent cessation of the supplement may then leave the child worse off (Chang et al., 2013). Most of the undernourished children in the current study had at least some of these risk factors, such as problems with breast feeding, late complementary feeding, low meal frequency and food refusal, which all have the potential to be modified via better advice and support at health facility level. However, few had all five risk factors present and half had only between one and three risks, so that any advice given would need to be customized, based on the child's needs and history rather than generic advice. In many supplementation trials ‘counselling’ is provided as the comparator arm on the assumption that such interventions are of low efficacy (Lelijveld et al., 2020), but in practice, the counselling provided is usually generic and delivered either by lay workers or very briefly (Lelijveld et al., 2020). There is now evidence from a systematic review that training health workers improves their practice and reported energy intake (Sunguya et al., 2013) and that training effects persist (Cunningham et al., 2019), although evidence of their impact on undernutrition itself is still lacking. There is a need for trials of sustainable interventions that focus on identifying and addressing individual risk exposure, complemented with socio‐economic and infrastructure interventions.

## 5 CONCLUSIONS

Almost all children in this study were exposed to factors associated with undernutrition, but those exposed to three or more risk factors were more likely to be undernourished. Most undernourished children were exposed to a variable number and combination of potentially modifiable risk factors, suggesting the need for individualized problem‐solving interventions.

## CONFLICTS OF INTEREST

The authors declare that they have no conflicts of interest.

## AUTHOR CONTRIBUTIONS

ANM and CMW developed the overall research plan, ANM collected the data and performed statistical analysis with input from CMW and ALG. ANM and CMW wrote the first draft. ANM, CMW, ALG and EWK reviewed the subsequent drafts. ANM had primary responsibility for the final content. All authors have read and approved the final manuscript.

## Supporting information


**Table S1:** Socio demographic and hygiene characteristics of healthy, wasted and stunted children
Table S2: Infant feeding, dietary factors and feeding practices by child nutrition status

## Data Availability

The data that support the findings of this study are available from the corresponding author upon reasonable request.
