# Supplementary material for: Prevalence and overlap of known undernutrition risk factors in children in Nairobi Kenya
Source: Matern Child Nutr. 2021 Aug 6;18(1):e13261. doi: 10.1111/mcn.13261 (PMC8710128; doi:10.1111/mcn.13261)
Supplement: Supplementary file 1 — Table S1: Socio demographic and hygiene characteristics of healthy, wasted and stunted children Table S2: Infant feeding, dietary factors and feeding practices by child nutrition status [file MCN-18-e13261-s001.docx]

Supplementary table 1: Socio demographic and hygiene characteristics of healthy, wasted and stunted children

|  | **Healthy (n=172)** | **Wasted** ^†^ **(n=92)** | **Stunted only (n=133)** |
| --- | --- | --- | --- |
| **Number of children under 5** |  |  |  |
| One child | 142 (83.5) | 67 (72.8) | 93 (69.6) |
| More than one child | 28 (16.5) | 25 (27.2) | 40 (30.1) |
| P chi square compared to healthy |  | 0.040 | 0.005 |
| **Mothers education** |  |  |  |
| Primary and below | 61 (35.5) | 37 (40.2) | 62 (47.0) |
| Secondary and above | 111 (64.5) | 55 (59.8) | 70 (53.0) |
| P chi square compared to healthy |  | 0.446 | 0.043 |
| **Father’s education** |  |  |  |
| Primary and below | 34 (21.0) | 21 (26.6) | 36 (31.3) |
| Secondary or higher | 128 (79.0) | 58 (73.4) | 79 (68.7) |
| P chi square compared to healthy |  | 0.331 | 0.052 |
| **Water source** |  |  |  |
| Piped into house | 17 (9.9) | 22 (23.9) | 23 (17.3) |
| Public tap | 155 (90.1) | 70 (76.1) | 110 (82.7) |
| P chi square compared to healthy |  | 0.002 | 0.057 |
| **Toilet ownership** |  |  |  |
| Shared | 138 (80.7) | 74 (80.4) | 116 (87.2) |
| Owned by household | 33 (19.3) | 18 (19.6) | 17 (12.8) |
| P chi square compared to healthy |  | 0.958 | 0.128 |
| **Asset ownership (TV/Radio)** |  |  |  |
| Does not own | 13 (7.6) | 9 (9.8) | 23 (17.3) |
| Owns one or both | 158 (92.4) | 83 (90.2) | 110 (82.7) |
| P chi square compared to healthy |  | 0.542 | 0.009 |
| **Infant feeding** |  |  |  |
| **Introduction of complementary foods** |  |  |  |
| Timely introduction | 133 (77.3) | 61 (66.3) | 85 (65.4) |
| Early/Late onset CF (>6m) | 39 (22.7) | 31 (33.7) | 45 (34.6) |
| P chi square compared to healthy |  | 0.053 | 0.022 |
| **Breastfeeding Status** |  |  |  |
| Breastfeeding | 163 (94.8) | 84 (91.3) | 116 (87.2) |
| Not breastfeeding | 9 (5.2) | 8 (8.7) | 17 (12.8) |
| P chi square compared to healthy |  | 0.275 | 0.01 |

†Includes children who are both wasted and stunted

Supplementary table 2: Infant feeding, dietary factors and feeding practices by child nutrition status

| **Care practices** | **Healthy (n=172)** | **Wasted^†^ (n=92)** | **Stunted (n=133)** |
| --- | --- | --- | --- |
| **Feeding Frequency** |  |  |  |
| Adequate | 49 (28.5) | 22 (24.2) | 38 (29.7) |
| Low Feeding frequency | 123 (71.5) | 69 (75.8) | 90 (70.3) |
| P Compared to healthy |  | 0.45 | 0.82 |
| **Total Dietary Risks** |  |  |  |
| No risks | 5 (2.9) | 7 (7.6) | 6 (4.6) |
| 1 or more | 167 (97.1) | 85 (92.4) | 124 (95.4) |
| P Compared to healthy |  | 0.08 | 0.43 |
| **Eating and feeding behavior (EFB)** |  |  |  |
| **Avidity** |  |  |  |
| Low avidity | 27 (15.7) | 24 (26.1) | 33 (24.8) |
| High avidity | 145 (84.3) | 68 (73.9) | 100 (75.2) |
| P Compared to healthy |  | 0.042 | 0.047 |
| **Food refusal** |  |  |  |
| High food refusal | 6 (3.9) | 16 (23.5) | 19 (18.8) |
| Low | 146 (96.1) | 52 (76.5) | 82 (81.2) |
| P Compared to healthy |  | <0.001 | <0.001 |
| **Force feeding** |  |  |  |
| High force feeding | 30 (20.5) | 27 (38.6) | 34 (33.0) |
| Low | 116 (79.5) | 43 (61.4) | 69 (67.0) |
| P Compared to healthy |  | 0.005 | 0.027 |
| **Total EFB risks** |  |  |  |
| No risks | 130 (75.6) | 47 (51.1) | 75 (56.4) |
| 1 or more | 42 (24.4) | 45 (48.9) | 58 (43.6) |
| P Compared to healthy |  | <0.001 | <0.001 |

†Includes children who are both wasted and stunted
